# Full Digital Workflow for Mandibular Ameloblastoma Management: Showcase for Technical Description

**DOI:** 10.3390/jcm12175526

**Published:** 2023-08-25

**Authors:** Vincenzo Abbate, Giulia Togo, Umberto Committeri, Fernando Zarone, Gilberto Sammartino, Alessandra Valletta, Andrea Elefante, Luigi Califano, Giovanni Dell’Aversana Orabona

**Affiliations:** 1 Maxillofacial Surgery Operative Unit, Department of Neurosciences, Reproductive and Odontostomatological Sciences, Federico II University of Naples, 80131 Naples, Italy; 2 Dentistry Unit, Department of Neurosciences, Reproductive and Odontostomatological Sciences, Federico II University of Naples, 80131 Naples, Italy; 3 Neuroradiology Unit, Department of Advance Biomedical Sciences, Federico II University of Naples, 80131 Naples, Italy

**Keywords:** ameloblastoma, dental prosthetic rehabilitation, fibula flap, CAD-CAM

## Abstract

This is a showcase for technical description of a full digital workflow aimed to reconstruct and prosthetically rehabilitate the mandible after surgical resection. The surgery was performed following a computer-aided design and computer-aided manufacturing (CAD-CAM) guided workflow, using 3D reconstruction of the mandible and the fibula. After 2 years, when the ossification of the flap was reached and verified by a computed tomography (CT) scan, surgery was performed using a two-step implant rehabilitation, with successful outcomes.

## 1. Introduction

Maxilla and mandible are critical components of the facial skeleton, with several functional and aesthetic attributes. Total or subtotal resection can lead to severe impairment of the patient’s quality of life. The goal of reconstructive surgery is to restore patient symmetry and functionality as close as possible to their premorbid state. This is particularly important because the jaws represent the only toothed portion of the skeleton, with multiple functions such as mastication, breathing, swallowing, speech and lip competency, located in a cosmetically demanding region of the head and neck district.

Ameloblastoma is a rare aggressive odontogenic epithelial tumor: it is a slow-growing but locally invasive benign neoplasm involving the mandible (80%) and maxilla [1]. The most common presentation for ameloblastoma is a painless swelling of the mandible, occasionally associated with tooth displacement.

Surgery is the gold standard treatment for ameloblastomas, but the type of resection depends on tumor size and location. According to Dell’Aversana Orabona et al. [2], gross total tumor resection may be considered the gold standard treatment for a large or recurrent lesion and includes en bloc resection with 1–2 cm bone margins and immediate bone reconstruction to help with speech and swallowing.

Several donor sites of vascularized bone free flaps for head and neck reconstruction have been described in the literature. The fibula free flap is considered one of the main surgical option for mandibular reconstruction after tumor resection [3].

Dental prosthetic rehabilitation of large maxillofacial defects using free tissue transfer and endosseous implants is considered the standard of care and the fibula flap provides favorable bone quality and quantity to receive and integrate dental implants to facilitate prosthetic rehabilitation [4,5,6]. Usually, the implants are placed after the oncological resection and reconstruction to facilitate a better positioning of the implants on the fibula flap and to facilitate better control of its vitality [7].

Until recently, the results of the surgical restoration relied on surgical skills and it was an operator-dependent procedure with unpredictable results. Today, the application of computer-aided design (CAD) and computer-aided manufacturing (CAM) in the medical field allows surgeons to plan cases virtually and create personalized surgical devices, reducing surgical time and minimizing the chance of failure during the reconstruction. The free fibula flap has some limitations due to the height and contour of the fibula, but the rise in VSP (virtual surgical planning) and the progression in the prototypization techniques of surgical guides and implants helps overcome the challenges of the procedure to maximize functional and aesthetic results.

This is a showcase for the technical description of a full digital workflow aimed to reconstruct and prosthetically rehabilitate the mandible after surgical resection.

## 2. Materials and Methods

**Figure 1:**
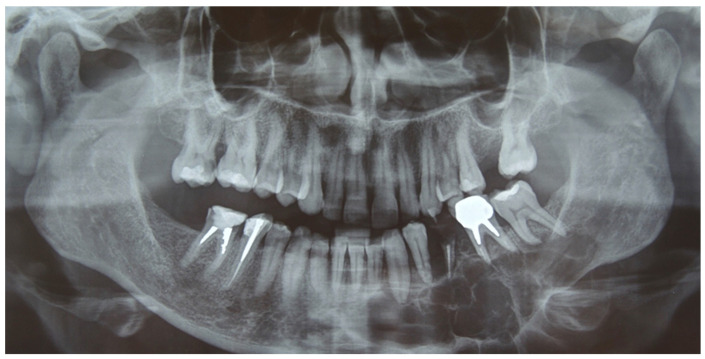
Preoperative Rx-OPT showing a multilocular radiolucency (from 3.2 to 3.8), suggesting an ameloblastoma.

A 41-year-old woman was admitted in our unit for an ameloblastoma of the left mandible. Clinical examination revealed a swelling of the alveolar region from 3.2 to 3.8 with irregular edges and firm consistency. The orthopantomography (OPT) and the head and neck computer tomography (CT) scan with thin slices of 1 mm showed an osteolytic lesion, with multilocular radiolucency extending from 3.2 to the ascending ramus of the left side (Figure 1).

Incisional biopsy of the lesion was performed and showed a solid ameloblastoma. A CT angiography of the lower extremities was performed to evaluate the vessels and bone for the FFF.

The patient underwent a partial mandibulectomy (chin, body and mandibular angle): a full digital workflow for the microsurgical reconstruction with free fibula flap and prosthetic implant rehabilitation is described below.

### 2.1. Preoperative Workflow

#### 2.1.1. Mandibular and Fibula Processing Data from DICOM to STL

**Figure 2:**
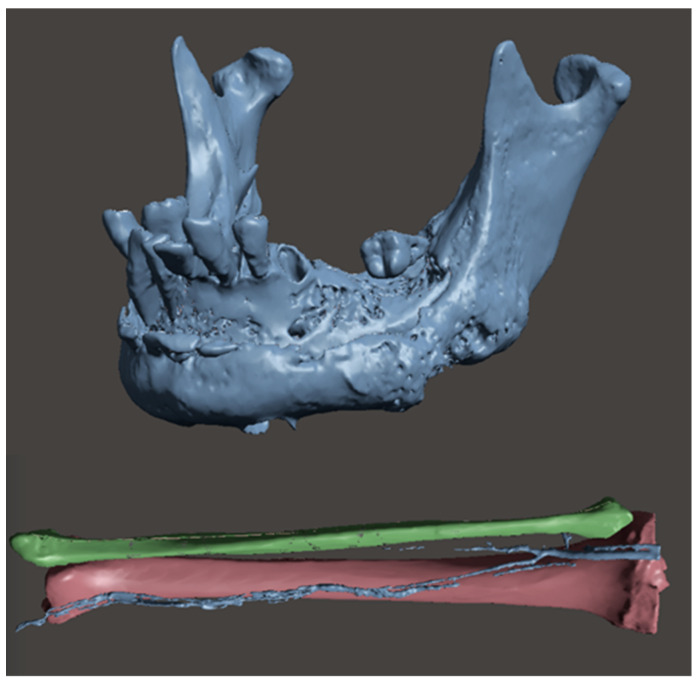
CT data acquisition of the mandible ad fibula.

CT data acquisition of the mandible and fibula were performed and were processed using Horos software (https://horosproject.org/). On the basis of the digital imaging (DICOM) data acquired from the CT scan, the mandible was reconstructed in 3D using InVesalius software (https://invesalius.github.io/) (Technology of Information Renato Archer Center of the Ministry of Science and Technology, Campinas, Brazil) to produce a standard triangulation language (STL) file of the patient’s mandibular and fibular bones (Figure 2).

#### 2.1.2. Meshmixer Processing

The STL file was initially uploaded into the open source software Meshmixer by Autodesk in San Rafael, CA. The lesion was then virtually resected using the “plane-cut” tool. Subsequently, the intact side of the mandible underwent processing with the “mirror” function, generating a virtual guide for the defective side. The goal was to replicate the pre-resection state as closely as possible by superimposing the 3D fibular image onto the mandibular defect, ensuring the best orientation.

**Figure 3:**
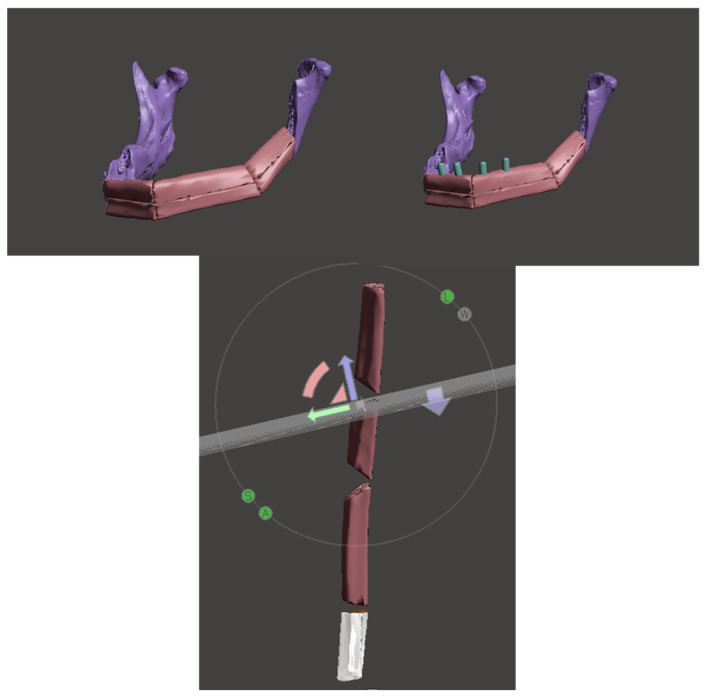
Virtual surgical planning of the mandibular resection and fibula osteotomy guides.

To achieve this, various tools such as “extrusion” and “thread” were utilized to model the guides according to the selected design. Measurements, including linear distance and gonial angle from the osteotomized portion of the mandible, were calculated through the “measure” function. These measurements were then applied to the fibular segment to create osteotomy guides for the specific bony portions required for mandibular reconstruction. Furthermore, the virtual design also entailed developing fibula osteotomy guides for any necessary bone divisions. In such cases, osteotomy planes were set and virtually cut for each aspect of the fibula (Figure 3).

Overall, the described process facilitated the precise planning of mandibular reconstruction by virtually creating guides and defining osteotomy points on the fibula, streamlining the surgical procedure and enhancing overall accuracy.

Through the “Boolean subtraction” tool, mandibular and fibular volumes were subtracted from the guide device to obtain the perfect fitting at the bone–guide interface.

#### 2.1.3. Rapid Prototyping

The digital models were printed in a stereolithography (SLA) 3D printer (Form 2, Formlabs, Somerville, MA, USA) with surgical guide resin (Formlabs) at a 0.1 mm printing resolution. After printing, the models were removed from the build platform and washed for 20 min in a Form Wash (Formlabs) filled with 99% isopropyl alcohol to clean the parts and remove the liquid resin. Then they were post-cured at 60 °C for 30 min in a Form Cure (Formlabs) to achieve biocompatibility and optimal mechanical properties.

**Figure 4:**
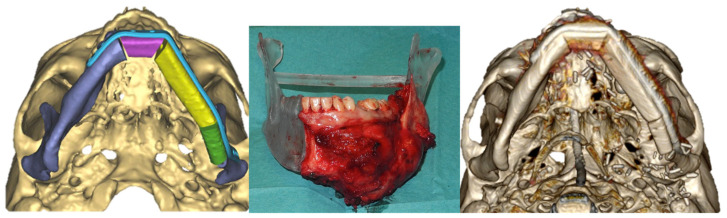
Mandibular resection inserted in the 3D printed model to verify the accuracy of the CAD-CAM guided presurgical planning and postoperative CT scan.

Prior to the surgery, the physic models were used to model the titanium reconstruction plates; then, the osteotomy surgical guides and the titanium plates underwent a sterilization using a low temperature hydrogen peroxide plasma technology (STER-RAD; Advanced Sterilization Products, Division of Ethicon US, LLC) (Figure 4).

### 2.2. Performing Surgery

The surgery was performed under general anesthesia. A mucosal incision from 3.8 to 4.3 was performed and after the jaw exposure, the surgical osteotomy guides were fixed to the bones with titanium screws (Synthes, West Chester, PA). Mandibular and fibula osteotomies were performed using a surgical saw and a piezoelectric device (Piezosurgery Plus, Mectron s.p.a. 2014). The bony segments were connected using the titanium plates designed and modeled on the digitally planned model. Microvascular anastomoses were performed inside the neck, the peroneal artery was anastomosed end-to-end to the facial artery, while meanwhile one of the peroneal vein was anastomosed end-to-end to a major tributary of the internal jugular vein. A skin paddle was used to cover the mucosal gap.

### 2.3. Postoperative Implant Rehabilitation

At 2 years of follow-up, a CT scan was performed to time the optimal placement of the implant rehabilitation.

Implant rehabilitation was carried out using a guided CAD-CAM technique to avoid interference with the reconstruction plate. The procedure was performed under local anesthesia: an intraoral incision in the buccal vestibule was performed and the alveolar ridge was exposed. Four endosseous implants (Tekka In-Kone) were positioned, as programmed in the virtual plan, through a dental- and crestal-supported surgical guide.

**Figure 5:**
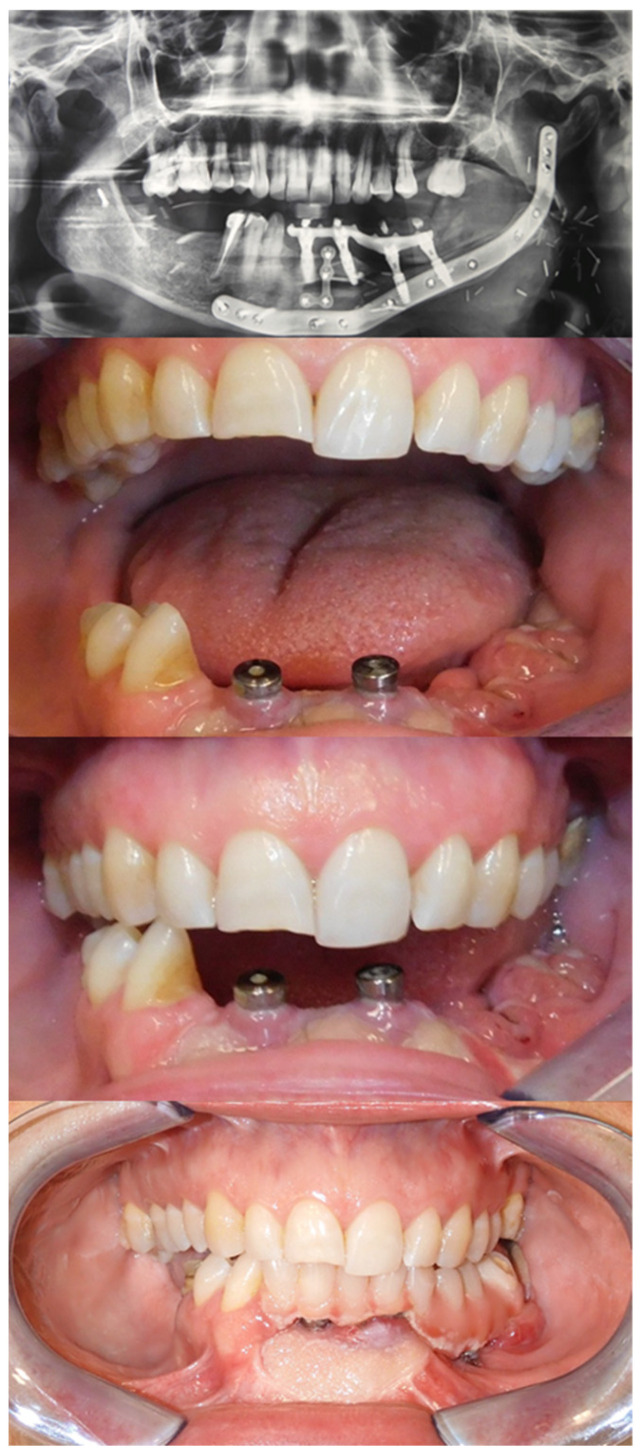
Dental implant rehabilitation after 2 years from surgery.

One month later, after a radiographic confirmation of osseointegration, the final implant-retained prosthesis was placed to complete the oral rehabilitation. There was no need for a flap thinning prior to the implant placement. (Figure 5).

## 3. Results

After obtaining the radical resection, the histological diagnosis confirmed the initial ameloblastoma suspect. A significant bone defect of 33 cm in length was identified and subsequently removed, followed by successful reconstruction. The surgery and immediate postsurgical care were uneventful, and no complications such as allergies or infections were observed.

The removal of plates and screws was not performed. The patient did not report any discomfort related to the plates and screws; moreover, the CAD/CAM workflow allowed the implants to be positioned without interfering with the reconstruction plate and its screws.

The mandibular reconstruction procedure achieved a positive outcome, with the digitally planned 3D models demonstrating excellent alignment with the final surgical results. To produce the model and guide, the total cost incurred was about EUR 4.6; meanwhile, the complete service for the start-up process of a CAD/CAM system costs between EUR 4000 and EUR 6000. Overall, the surgical intervention and reconstruction proved successful, providing an effective solution for the ameloblastoma-related bone defect and the expenses associated with manufacturing the 3D models and guides were reasonable.

## 4. Discussion

The reconstruction of mandibular defects is a complex procedure due to the anatomic and functional features of the bone.

The fibula free flap (FFF) has become the gold standard for surgical reconstruction of mandibular bony defects since Hidalgo first used it in 1989 [8].

The FFF is the most used vascularized bone graft (VBG) used in orofacial reconstructions because it provides adequate bone length, long vascular pedicle and bicortical architecture, increasing primary implant fixation. Implant failure rates in fibula free flaps are higher compared to the native mandibular bone; in any case, a success rate exceeding 91% has been reported [9].

The main difficulty of a FFF in a mandibular reconstruction is represented by the modeling and reshaping of the fibula to achieve proper volume and height for the future dental implant rehabilitation. Another critical step is the intra-surgical correct modeling of the titanium plate, in order to avoid a plate breakage after improper adjustments and reshaping. Since Hirsch et al. [10] first described the computer-assisted surgery (CAS) or computer-aided design and computer-aided manufacturing (CAD/CAM) for mandibular reconstruction in 2009, this technology has gained popularity and has been applied successfully even for the challenging secondary mandibular reconstruction.

The advantages of surgical CAD/CAM reconstructive procedures include ideal presurgical planning for tumor resection and surgical reproducibility for site and orientation osteotomies. The main disadvantages are the cost and the product delivery time.

As showed by this case, CAD/CAM prototypization allowed modeling of the titanium plate prior to surgery, minimizing the stress and the bending fatigue of the plate and reducing the risk of fracture. Moreover, the digital creation of osteotomy guides offered a good bone-to-bone contact between the distal fibula segment and the residual mandible, maximizing the post-reconstruction facial symmetry. The digital surgical planning offers a noticeable reduction in operation time, reducing both the blood loss and the risks of ischemia of the fibula flap [11].

In order to reduce the ischemic time of the fibular flap, the donor pedicle should not be dissected from the lower leg until the harvested fibula has already been shaped and the recipient vessel prepared [12].

Dental implants are one of the important factors involved in the multidisciplinary rehabilitation of patients who have undergone a surgical resection of the maxillofacial district. Improving the optimal aesthetic and functional outcomes for patients with mandible ameloblastoma can be achieved using dental implants. A successful dental restoration can be more challenging on these particular types of patients because of the surgical resection of bone and the damage of the soft tissue, mostly the oral mucosa.

A functional and stable prosthetic rehabilitation after tumor resection can only be achieved using osseointegrated implants; due to the retention of bone height, they provide a reliable long-term stability, whereas removable partial dentures retained by clasps to the remaining teeth are associated with gradual bone loss.

A possible drawback of the fibular flap is a relative lack of bone height, but this limitation can be overcome by double-barreling the fibular flap for the mandibular reconstruction.

The CAD/CAM workflow allows for a highly accurate implant placement, allowing the insertion of implants to ensure maximum resistance to masticatory forces based on the thickness of FFF and minimizing the angular deviation between the central axes of the planned and final position of the implant.

As reported by Ch’ng et al., it is difficult to define an appropriate protocol for the placement of implants in patients with head and neck cancer [9] and most of the previous studies reported a very low rate of dental implant placement in mandibular reconstruction [13]. Moreover, not every patient is eligible for FFF: donor site availability, morbidity, ease of flap dissection and the status of the recipient vessels in the neck, as well as the patient’s overall medical condition may also influence the final decision. As well, not every patient can be a candidate for oral rehabilitation because of factors such as oral hygiene, prognosis and patient cooperation. In this case, the patient agreed to reposition the implants 2 years after surgery.

Several factors must be considered for the timing of a postsurgical dental implant rehabilitation in a VBG; among these, the donor site morbidity is paramount.

Placing the osseointegrated implants with a 6–24 months delay after the reconstructive surgery allows for optimum control of the fibula flap vitality, reducing the possibility of a flap failure after the implant rehabilitation.

A proper healing time before the implant’s placement ensures a good bone regeneration, which should be evaluated by an OPT and should include an appropriate remodeling and adaptation of the intraoral soft tissue surrounding the reconstructed segment [14]. Hence, a delayed approach allows for a far more comprehensive assessment of the disease status, oral function and patient motivation, as well as more precise prosthetic planning [15].

## 5. Conclusions

The fibula free flap is considered to be one of the main surgical options for mandibular reconstruction after large bone resections, especially in cases of a benign lesion, such as ameloblastomas. The CAD-CAM presurgical planning can provide optimal bone healing, ensuring the vitality of the free flap and allowing for the anatomical and functional rehabilitation of the mandible, using dental implants, and improving the patient’s quality of life. Appropriate timing of the placement of dental implants is critical to guarantee the successful outcome in patients who need this type of reconstruction.

## Data Availability

Data sharing is not applicable to this article.
